# Quantitative Assessment of the Effects of Oxidants on Antigen-Antibody Binding* In Vitro*

**DOI:** 10.1155/2016/1480463

**Published:** 2016-05-24

**Authors:** Shuang Han, Guanyu Wang, Naijin Xu, Hui Liu

**Affiliations:** ^1^College of Medical Laboratory, Dalian Medical University, Dalian 116044, China; ^2^Second Affiliated Hospital of Dalian Medical University, Dalian 116023, China

## Abstract

*Objective.* We quantitatively assessed the influence of oxidants on antigen-antibody-binding activity.* Methods.* We used several immunological detection methods, including precipitation reactions, agglutination reactions, and enzyme immunoassays, to determine antibody activity. The oxidation-reduction potential was measured in order to determine total serum antioxidant capacity.* Results.* Certain concentrations of oxidants resulted in significant inhibition of antibody activity but had little influence on total serum antioxidant capacity.* Conclusions.* Oxidants had a significant influence on interactions between antigen and antibody, but minimal effect on the peptide of the antibody molecule.

## 1. Introduction

Antigen-antibody response constitutes a basic immunological reaction and describes a specific response between antigen and antibody [1]. Antibodies are important to humoral immunity, and many biological activities, including bacteriolysis, sterilization, and toxin neutralization, are based on antigen-antibody response. Immunomodulation is also an antigen-antibody response to humoral immune activation* in vivo*, and any damage to the antibody can affect immune function [2]. In this study, we focused on quantitative assessment of the influence of oxidants on antigen-antibody interaction by determining total serum antioxidant capacity and the effects of oxidative stress on the immune system.

Oxidative stress occurs during aging in humans due to an increase in free radical generation and/or a reduction in clearance capacity, which causes disorders to oxidation and antioxidant systems [3, 4]. High concentrations of reactive oxygen species (ROS) can increase the risk of DNA damage, lipid peroxidation, and protein carbonylation reactions [5]. Studies showed that ROS plays an important role in regulating immune response [6] and that ROS is an important indicator of inflammation and may also be a diagnostic marker for interactions between the immune system and pathogens [7]. The relationship between oxidative stress and immune response has been studied in clinical medicine at the molecular level, and the etiology of human diseases, such as cardiovascular disease, cancer, and diabetes, has been associated with oxidative stress and the immune response [3, 7, 8]. However, research on the influence of oxidants on antigen-antibody-binding activity has been rarely reported. Here, we simulated the process of oxidative stress* in vivo* using three oxidants (potassium permanganate, iodine, and hydrogen peroxide) in order to detect their effect on serum incubated at 37°C for 30 min [9, 10]. Three immunological detection methods, including precipitation reactions, agglutination reactions, and enzyme immunoassays, were employed to determine antigen-antibody-binding activity and to assess the effects of oxidative stress on total serum antioxidant capacity in the immune system.

## 2. Materials and Methods

### 2.1. Precipitation Reaction (Double-Diffusion Test)

#### 2.1.1. Treatment of Test Specimens

Oxidants (20 mM potassium permanganate solution, 20 mM iodine solution, and 50 mM hydrogen peroxide solution) were diluted at a 1 : 1 ratio using distilled water to obtain five concentrations. The test specimen (a rabbit polyclonal anti-human whole-serum antibody) was diluted with saline at a 1 : 1 ration, followed by mixing with the oxidant dilutions at 1 : 1 ratios. The mixed samples and saline (control) were incubated at 37°C for 30 min. The pH of the test specimen was determined, and if the pH was outside of 7.2 to 7.5, new reagents were prepared.

#### 2.1.2. Double-Diffusion Test

Seven holes were inserted into the prepared agar plate and 20 *μ*L of healthy human serum diluted with normal saline at a 1 : 4 ratio was added to the central hole using a micropipette. Next, 20 *μ*L of antibody was added to the other six holes. The gel plate was then placed in an incubator at 37°C for 24 h to observe the precipitation line.

#### 2.1.3. Precipitation Intensity

The precipitation line was scored as 0, 1+, 2+, or 3+ according to precipitation intensity. Fifteen parallel tests were performed to calculate the average reaction intensity at different oxidant concentrations, and the average reaction intensity in the control group (i.e., saline) was also determined. If the reaction intensity of an oxidant was less than that of the control group or equal to half of that observed in the control group, this indicated that the concentration of oxidizing agent was high enough to produce an inhibitory effect on antibody activity. When the reaction intensity in the control group was half of the average reaction intensity, we defined this value as ID_50_ (half-inhibition dose). The closest value to the upper and lower reaction intensities was obtained, and the corresponding concentration of oxidant was used to obtain the reaction equation. ID_50_ was included in the equation and corresponded to the 50% inhibitory concentration.

For example, if the average reaction intensity for the control well in a potassium permanganate reaction was 2.3 and the reaction intensity corresponding to ID_50_ concentration should be 1.15, the reaction intensity was between 0.73 (corresponding to 2.5 mM of potassium permanganate) and 1.6 (corresponding to 1.25 mM of potassium permanganate). A linear equation through these two values can be expressed as *y* = −0.696*x* + 2.47, where *y* represents the reaction intensity and *x* represents the concentration. When *y* = 1.15 and *x* = 1.9, ID_50_ concentration of potassium permanganate is 1.9 mM.

### 2.2. Agglutination Reaction

#### 2.2.1. Treatment of Test Specimens

Here, 20 mM potassium permanganate solution and 20 mM iodine solution were diluted at a 1 : 3 ratio with distilled water in order to prepare five dilutions. Then, 50 mM hydrogen peroxide solution was similarly diluted at a 1 : 1 ratio to obtain five dilutions. The test specimens (blood type O healthy human serum and antibodies to blood types A and B) were then mixed with the oxidant dilutions and saline (control) at a 1 : 1 ratio, and the mixtures were incubated at 37°C for 30 min. The pH of the test specimen was determined, and if the pH was outside of 7.2 to 7.5, new reagents were prepared.

#### 2.2.2. Agglutination Reaction

In 10 small test tubes, the treated samples were diluted 10-fold at 1 : 1 ratios, followed by addition of 0.1 mL 2% red blood cells (type A) to each tube. The tubes were shaken to make sure that the mixtures were homogeneous, and all the tubes were centrifuged at 2000 rpm for 2 min.

#### 2.2.3. Interpretation of Results

Agglutination (100%) was scored as 4+, 75% agglutination as 3+, 50% agglutination as 2+, and 25% agglutination as 1+. In the case of minimal agglutination, the background turbidity was scored as W+. If no agglutination and no hemolysis were observed, all cells were defined as free and scored as 0.

**Figure 1:**
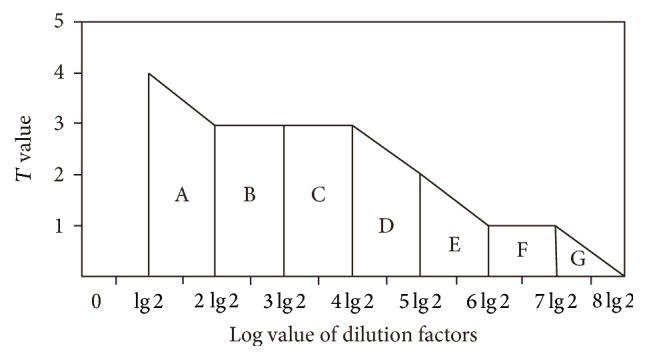
Schematic diagram of the degree of aggregation calculated as the total area under the plotted line.

To simplify calculations, serum-dilution factors (1 : 2, 1 : 4, 1 : 8, etc.) were transformed into a logarithmic scale (lg 2, 2 lg 2, 3 lg 2, etc.). The square of the oxidant concentration and the corresponding aggregation-response intensity was determined as a measure of the effects of oxidants on antibody activity in the agglutination reaction (Figure 1):

(1) Ssquare=T2+2×T4+T8+T16+T32+T64+T128+T256,

where *T* represents the aggregation strength in a dilution. Changes in *T* were arranged from the largest to the smallest; therefore, all of the *T* values should be considered together. The area under the curve was a better indicator for evaluating the effects of oxidant concentration on antibody activity as compared to *T* value.

For example, the area, *A*, and dilution factors 2 and 4 could be regarded as the area of the trapezoid bounded by lg 2, 2 lg 2, *T*2, and *T*4, and where the square (*S*) can be calculated as follows:

(2) $$\begin{array}{c} S_{A}=\left(\;T2+T4\;\right)\times \frac{\left(2\;lg\;2-lg\;2\right)}{2}. \end{array}$$

For this series of dilutions, the dilution interval is constant, meaning that 1/2 (lg 2) is a constant that can be ignored. Therefore, *S*_*A*_ = *T*2 + *T*4 and the total area is equal to

(3) SSA+SB+SC+SD+SE+SF+SG=T2+T4+T4+T8+T8+T16+T16+T32+T32+T64+T64+T128+T128+T256=T2+2×T4+T8+T16+T32+T64+T128+T256.

*S*_NS_ (NS represents normal saline) of the control group is calculated in the same way. We used the concentration of the oxidant corresponding to half of *S*_NS_ as ID_50_ concentration, which had a significant inhibitory effect on the activity of the antibody. This was calculated through the equation of precipitation experiment.

### 2.3. Enzyme-Linked Immunosorbent Assay (ELISA)

#### 2.3.1. Treatment of the Test Specimens

The procedures were similar to those described in Section 2.1.1. The test specimen consisted of healthy human mixed serum with an antibody to hepatitis B surface antigen. The pH of the test specimen was determined, and if the pH was outside of 7.2 to 7.5, new reagents were prepared.

#### 2.3.2. Determination of Hepatitis B Surface Antibody

The double-antigen sandwich ELISA method was used according to the manufacturer's instructions associated with the Diagnostic Kit for Antibody to Hepatitis B Surface Antigen.

#### 2.3.3. Processing of Results

Optical density (OD) was measured to assess antibody activity, with higher OD values indicating higher antibody activity. The OD value for the blank plate was 0.082 and was eliminated from the next calculation. We used the concentration of the oxidant corresponding to half of OD_NS_ as ID_50_ concentration that exerted a significant inhibitory effect on the activity of the antibody. The calculation method was the same as that used in the precipitation experiment.

### 2.4. Total Serum Antioxidant Capacity

#### 2.4.1. Preparation of Oxidants under ID_50_ Concentrations

For the precipitation, agglutination, and ELISA reactions, ID_50_ concentrations of three oxidants could be obtained; oxidants under ID_50_ concentrations should be prepared.

#### 2.4.2. Treatment of the Test Specimens

Serum specimens were randomly collected from 20 healthy people (10 males and 10 females). The test specimens (200 *μ*L serum + 200 *μ*L oxidant under ID_50_ concentration), the negative control (200 *μ*L serum + 200 *μ*L saline), and the positive control (200 *μ*L phosphate-buffered saline (PBS) buffer + 200 *μ*L oxidant under ID_50_ concentration) were mixed at 1 : 1 ratios, respectively, and all samples were incubated for 30 min at 37°C. The pH of the test specimen was determined, and if the pH was outside of 7.2 to 7.5, new reagents were prepared.

#### 2.4.3. Detection of the Oxidation-Reduction Potential (ORP)

A micro Pt/AgCl electrode was immersed into the test samples; then a reading was recorded in millivolts (mV) after the ORP value was stable for 5 s. The operation was performed in an anaerobic glove box, and the electrode was cleaned thoroughly after each measurement. The ORP is calculated according to the Nernst equation:

(4) $$\begin{array}{c} ORP\left(\;E\;\right)=E_{0}-\frac{RT}{nF}\times \ln\frac{\left[reductant\right]}{\left[oxidant\right]}, \end{array}$$

where *R* and *F* are constants, *T* represents the temperature in kelvin, and *E*_0_ is the standard potential for a redox system. ORP is related to both the total concentrations of the reductants and the total concentrations of the oxidants in a particular system [11, 12] and offers an overall measurement of redox status in a biological specimen.

### 2.5. Polyacrylamide Gel Electrophoresis (PAGE)

PAGE functions as molecular sieve and is capable of separating different proteins [13]. In this study, we performed PAGE experiments with test specimens treated by oxidants (saline for the control group). By observing band changes between the specimen and control groups, we were able to determine whether oxidizing agents changed the structure of antibodies or the peptide chains from denatured or hydrolyzed antibodies. Nondenaturing separation and stacking gels were established at 12% and 6%, respectively, and test samples were treated similar to the methods described in Section 2.4.3. Gels were electrophoresed for 1 h, followed by staining with Coomassie Brilliant Blue (CBB) and observation of band changes in the experimental and control groups.

#### 2.5.1. Electrophoresis

Gel electrophoresis was run for an hour. Following electrophoresis, the gel was stained with CBB and then was decolorized to observe the results. If oxidizing agents altered protein structure, this would be shown accordingly based on changes in molecular weight and position of the band on the gel. If the bands in experimental and control groups were consistent in their positions, this demonstrated that the oxidant did not cause a significant change in the structure of the protein.

### 2.6. Statistical Analysis

SPSS 17.0 statistical software (SPSS, Inc., Chicago, IL, USA) was used for statistical analysis of experimental data. The results of detection using clinical specimens were represented as the mean ± standard deviation, and the oxidant and control groups were compared using the *t*-test. Statistical analysis with *α* = 0.05 was used as the inspection level.

## 3. Results

### 3.1. Precipitation Reaction

**Figure 2:**
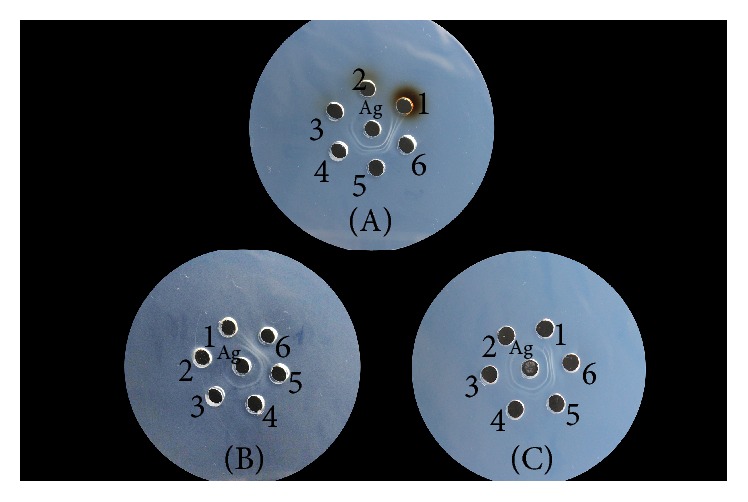
Effect of potassium permanganate, iodine, and hydrogen peroxide on the activity of the antibody in the precipitation reaction. (A): numbers 1, 2, 3, 4, and 5 correspond to the concentrations of potassium permanganate 20 mM, 10 mM, 5 mM, 2.5 mM, and 1.25 mM, respectively. Number 6 is the control group. (B): numbers 1, 2, 3, 4, and 5 correspond to the concentrations of iodine 20 mM, 10 mM, 5 mM, 2.5 mM, and 1.25 mM, respectively. Number 6 is the control group. (C): numbers 1, 2, 3, 4, and 5 correspond to the concentrations of hydrogen peroxide 50 mM, 25 mM, 12.5 mM, 6.25 mM, and 3.125 mM, respectively. Number 6 is the control group.

Fifteen parallel tests were performed, with the results indicating that potassium permanganate, iodine, and hydrogen peroxide all exhibited inhibitory effects on antibody activity in the precipitation reactions. ID_50_ concentrations of the oxidants were 1.9 mM, 2.96 mM, and 15.9 mM, respectively, as shown in Figure 2 and Table 1.

### 3.2. Agglutination Reaction

Potassium permanganate, iodine, and hydrogen peroxide all exhibited inhibitory effects on antibody activity in the agglutination reaction. ID_50_ concentration is shown in Table 2.

### 3.3. Enzyme Immunoassay

**Figure 3:**
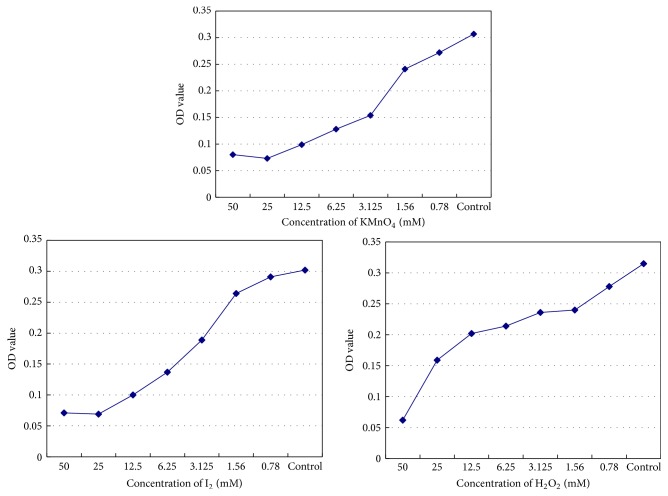
Effect of potassium permanganate, iodine, and hydrogen peroxide on hepatitis B surface antibody activity.

Potassium permanganate, iodine, and hydrogen peroxide all exhibited inhibitory effects on antibody activity according to ELISA. ID_50_ concentrations of each oxidant were 2.40 mM, 3.06 mM, and 13.5 mM, respectively (Figure 3).

### 3.4. Determination of Total Serum Antioxidant Capacity

We observed that the ORP values of the oxidant control (potassium permanganate, iodine, and hydrogen peroxide controls were 558.65 mv, 542.25 mv, and 536.85 mv, resp.) were far greater than the experimental and saline control groups. We then compared the experimental groups with the control group and found *P* > 0.05 in all cases. According to the inspection level of *α* = 0.05, our results showed no statistical significance, indicating that the total serum antioxidant capacity had not changed significantly (Table 3).

### 3.5. Serum Protein PAGE

**Figure 4:**
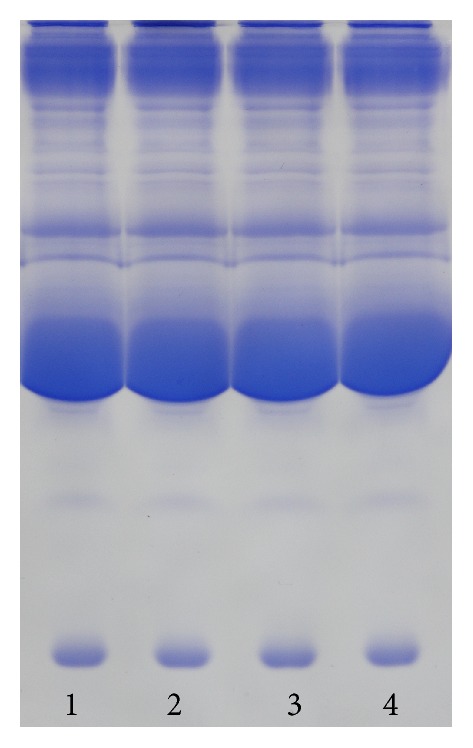
Results of serum protein gel electrophoresis. Numbers 1, 2, and 3 correspond to the ID_50_ concentrations of potassium permanganate, iodine, and hydrogen peroxide, respectively. Number 4 is the control group.

Oxidants under their ID_50_ concentrations were added to serum samples, followed by PAGE analysis. The results indicated that the bands associated with the oxidant and control groups were in the same positions on the gel, illustrating that each oxidant at a specific concentration did not alter protein structure (Figure 4).

## 4. Discussion

Precipitation reactions involve formation of an insoluble antigen-antibody complex under appropriate conditions. The double-diffusion test is a common method used for identification of antigen-antibody reactions and involves the diffusion of a soluble antigen and corresponding antibody in agar to form a line of antigen-antibody precipitate in the correct proportion. By observing the position of the formed precipitation line, shape, and contrast relationship, the relative amounts of antigen-antibody complex can be estimated [14]. The double-diffusion test performed in our study showed that potassium permanganate, iodine, and hydrogen peroxide all inhibited serum antibody activity, and that the ID_50_ concentrations were 1.9 mM, 2.96 mM, and 15.9 mM, respectively.

Agglutination reactions allow observation of particle aggregation of red blood cells or bacteria and other antigen or surface antigen particles coated with carriers in the presence of an antibody or complement. This method can be used for semiquantitative detection [15, 16], and in our study, this reaction was used to determine serum IgM antibody activity. The results demonstrated that potassium permanganate, iodine, and hydrogen peroxide inhibited antibody activity at ID_50_ concentrations of 2.58 mM, 3.83 mM, and 22.3 mM, respectively. Compared with results from the precipitation reaction, the agglutination test is highly sensitive.

Enzyme immunoassay technology combines the specificity of the antigen-antibody reaction with that of an efficient enzymatic reaction and can be used to fix antigen or antibody for quantitative or qualitative analysis [17]. It is highly sensitive and specific, offers good accuracy, simplicity, environmental safety, and reagent stability, and is a leading clinical immunoassay technology. We used ELISA to determine hepatitis B surface antibody activity, resulting in increased sensitivity, improved quantitative analysis, and higher accuracy as compared with the precipitation and agglutination techniques. Our results using this method showed that potassium permanganate, iodine, and hydrogen peroxide all exhibited inhibitory effects on antibody activity at ID_50_ concentrations of 2.40 mM, 3.06 mM, and 13.5 mM, respectively.

Here, we evaluated a polyclonal antibody by precipitation reaction, given that monoclonal antibodies are not detectable by this method. Since polyclonal antibodies and antigen surface epitopes were more easily cross-linked into a network structure, this enables precipitation reactions. The IgM antibody plays a major role in agglutination reactions. When double-antigen ELISA was used to detect hepatitis B surface antibodies, we observed that the quantitative analysis of the IgG antibody was more accurate and sensitive relative to other techniques. The three methods used here complement each other and verified the effects of oxidants on various types of antibodies. Our results indicated that oxidants had an inhibitory influence on antibody activity.

Previous reports focused on the effects of oxidative stress on immune complex formation; however, minimal research has been carried out concerning the effects of oxidants on antibody activity. Here, we performed precipitation and agglutination reactions and ELISA to determine the effects of oxidants on antibody activity, with each confirming that specific oxidant concentrations exhibited significant inhibitory effects on antibody activity. When oxidants at their ID_50_ concentrations were added to serum to detect total serum antioxidant capacity, we observed that oxidant concentration had minimal influence on total serum antioxidant capacity.

The results of PAGE analysis showed that the primary structure of each protein was not altered at oxidant ID_50_ concentrations; therefore, future* in vivo* experiments may benefit from our* in vitro* results. Antigen-antibody reactions involve complementary binding between an antigen molecule surface epitope and hypervariable regions of antibody molecules [18]. This is determined both by the spatial configuration of antigen and antibody molecules and by the combination of antigen epitope and molecular surface grooves located in antibody hypervariable regions [1]. Electrostatic attraction, Van der Waals forces, hydrogen bonding, and hydrophobic interactions between antigen and antibody promote the antigen-antibody interactions and subsequent formation of the antigen-antibody complex.

In conclusion, our results suggested that oxidants altered the secondary or tertiary structure of antibodies, possibly resulting in exposure of hydrophobic groups at the antibody surface, adversely affecting antigen-antibody interactions. Our* in vitro* findings demonstrated that the oxidants at ID_50_ concentrations did not destroy the primary structure of the antibody, which is consistent with normal biological functions under oxidative stress, which is an important cause of decreased antibody activity. Although we observed minimal influence by oxidants on total serum antioxidant capacity, they significantly affected antigen-antibody binding, which can adversely affect immune function, initiate immune dysfunction and decreased resistance to infection, and possibly lead to bacterial, viral, fungal, or other infections, or possibly cancer [19, 20]. Further study is needed to illustrate the detailed mechanisms.

## Figures and Tables

**Table 1:** Results of oxidants in the precipitation reaction.

Oxidants	Concentrations (mM)	Maximum	Minimum	Standard deviation	Mean	ID_50_ concentration (mM)
KMnO_4_	20	0	0	0	0	1.9
	10	0	0	0	0	
	5	1	0	0.37	0.2	
	2.5	1	0	0.46	0.73	
	1.25	2	1	0.51	1.6	
	*Control group*	*3*	*2*	*0.49*	*2.3*	

I_2_	20	0	0	0	0	2.96
	10	0	0	0	0	
	5	1	0	0.44	0.63	
	2.5	2	1	0.49	1.33	
	1.25	3	1	0.70	1.93	
	*Control group*	*3*	*2*	*0.51*	*2.4*	

H_2_O_2_	200	1	0	0.35	0.53	15.9
	100	1	0	0.37	0.8	
	50	2	1	0.51	1.4	
	25	3	1	0.64	2.13	
	12.5	3	2	0.49	2.33	
	*Control group*	*3*	*2*	*0.52*	*2.47*	

**Table 2:** Results of oxidants in the agglutination reaction.

Oxidants	Concentrations (mM)	Average intensity of the reaction (area)	ID_50_ concentration (mM)
KMnO_4_	20	11	2.58
	5	15	
	1.25	22	
	0.3125	32	
	0.078	36	
	*Control group*	*39*	

I_2_	20	14	3.83
	5	18	
	1.25	26	
	0.3125	33	
	0.078	36	
	*Control group*	*41*	

H_2_O_2_	50	17	22.3
	25	20	
	12.5	27	
	6.25	36	
	3.125	39	
	*Control group*	*43*	

**Table 3:** Comparison of total antioxidant capacity before and after serum treatment with oxidants.

Group	Mean (mV)	Standard deviation	*t*	*P* value
KMnO_4_	344.25	22.85	1.726	0.101
I_2_	344.35	25.16	1.047	0.308
H_2_O_2_	345.10	20.71	1.919	0.070

Control (saline)	342.40	21.05	—	—

*Note*. The ORP values of potassium permanganate, iodine, and hydrogen peroxide controls were 558.65 mv, 542.25 mv, and 536.85 mv, respectively.
